# Ribosome Distribution in HeLa Cells during the Cell Cycle

**DOI:** 10.1371/journal.pone.0032820

**Published:** 2012-03-05

**Authors:** Yuan-Jhih Tsai, Hsing-I Lee, Alan Lin

**Affiliations:** 1 Institute of Genome Sciences, School of Life Sciences, National Yang-Ming University, Taipei, Taiwan; 2 Department of Dentistry, School of Dentistry, National Yang-Ming University, Taipei, Taiwan; Duke University Medical Center, United States of America

## Abstract

In this study, we employed a surface-specific antibody against the large ribosome subunit to investigate the distribution of ribosomes in cells during the cell cycle. The antibody, anti-L7n, was raised against an expansion segment (ES) peptide from the large subunit ribosomal protein L7, and its ribosome-surface specificity was evident from the positive immuno-reactivity of ribosome particles and the detection of 60 S immune-complex formation by an immuno-electron microscopy. Using immunofluorescent staining, we have microscopically revealed that ribosomes are dispersed in the cytoplasm of cells throughout all phases of the cell cycle, except at the G2 phase where ribosomes show a tendency to gather toward the nuclear envelope. The finding in G2 cells was confirmed by electron microscopy using a morphometric assay and paired t test. Furthermore, further observations have shown that ribosomes are not distributed immune-fluorescently with nuclear envelope markers including the nuclear pore complex, the integral membrane protein gp210, the inner membrane protein lamin B2, and the endoplasm reticulum membrane during cell division we propose that the mechanism associated with ribosome segregation into daughter cells could be independent of the processes of disassembly and reassembly of the nuclear envelope.

## Introduction

The biogenesis of a ribosome in the eukaryotic cell can be detected at the start cell cycle checkpoint [1], and it involves many aspects of the cellular machinery [2]. The energy requirement for ribosome genesis includes that needed for generating ribosomal components, processing and assembly, as well as their transportation [3], [4]. Current information on how ribosomesare distributed across the cell is very limited. There is much known about membrane-bound ribosomes, but practically nothing is known about the cytoplasmic distribution of free ribosomes. Previous research has suggested that ribosomes are redistributed such that they accumulate at the site of protein synthesis [5], [6], [7], implying that the ribosome population undergoes dynamic movement as required. To understand how a cell can command ribosome movement in cytoplasm to allow translation is thus of significant interest. Equally, how a cell distributes its ribosome particles during the cell cycle is also important. The latter issue would have a great impact on the survival of the daughter cells, which need an adequate number of ribosomes to ensure the synthesis of important proteins for future physiological events [8]. Up to the present, these issues have gone unstudied because, as suggested earlier [9], there is a lack of a good method for pinpointing and counting the ribosome particles in the cell. Obviously using immunofluorescent staining by a specific ribosome-surface antibody would be an ideal tool for localizing ribosome particles during cellular events, but such an antibody is quite difficult to produce.

Recently information on the structure of eukaryotic ribosome has greatly progressed [10], and the characteristics of the expansion segments (ES) of ribosomal rRNA and ribosomal peptides in eukaryotic ribosome have been gradually revealed [10], [11], [12], [13], [14]. These studies have suggested that the ES is often exposed on the surface of ribosome particle [10], [11], [12], [13], [14], [15]. Thus, the potential surface property of an ES might provide a useful means of generating a surface-specific antibody against eukaryotic ribosome particles. By this rationale, the ES peptide of the large subunit ribosomal protein L7 was selected for this purpose. The ES of L7, which consists of the first 54 amino acid residues, is derived from a phylogenic alignment, and is essential in eukaryotes [13]. Moreover, it has been shown that the ES is exposed on the surface of the large ribosome subunit [12], [13]. Accordingly, in this study, our first aim was to prepare an antibody against this ES peptide and established the surface property of this antibody. Next, we used this property to detect the cellular distribution of ribosomes during the cell cycle. Finally, we examined the possible involvement of the assembly/disassembly of the nuclear membrane in ribosome segregation.

## Results

### Characterization of the surface property of the anti-L7n antibody

**Figure 1:**
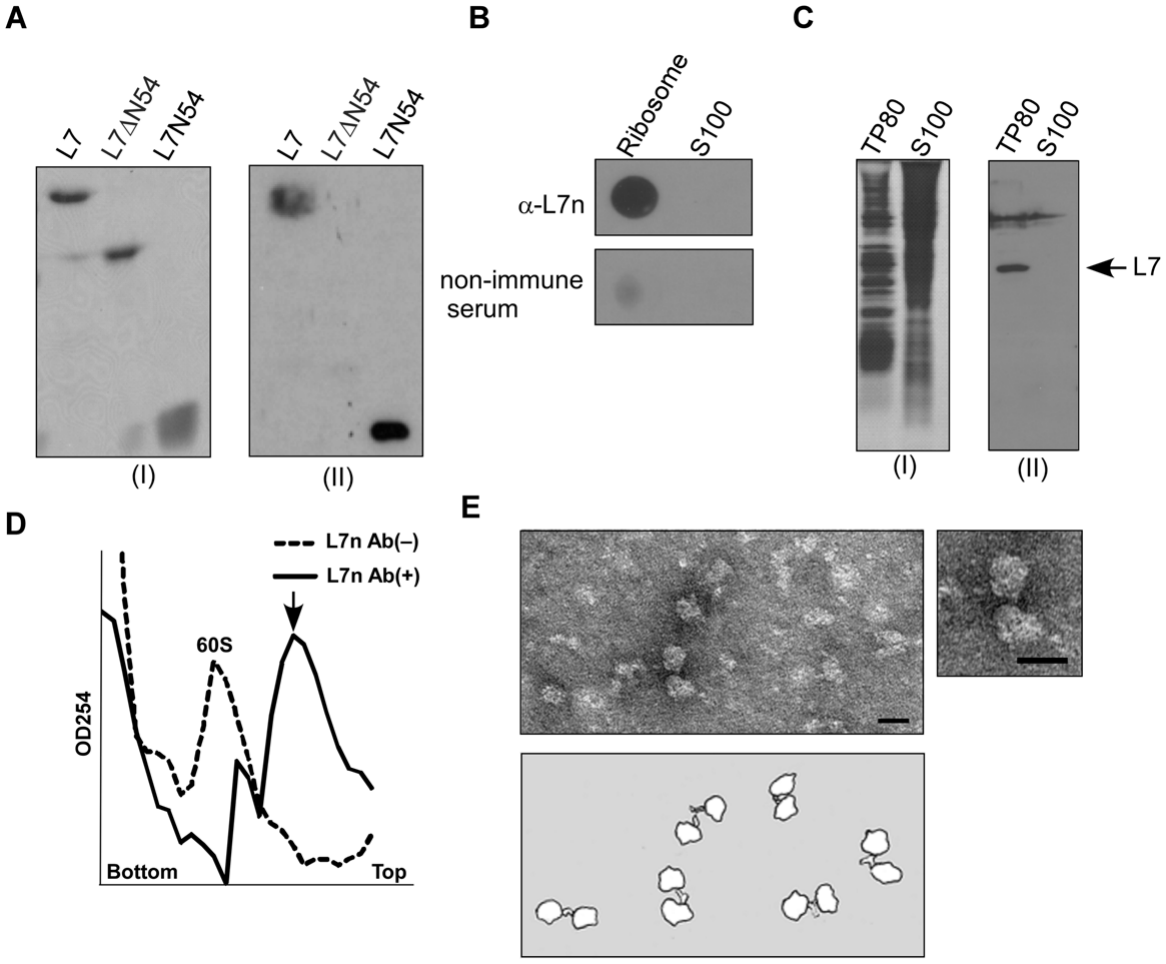
Characterization of the surface-specific properties of anti-L7n antibody against ribosome particles. (A) Immuno-reactivity of anti-L7n antibody against L7, L7ΔN54, and L7N54 analyzed by SDS-PAGE with Coomassie blue staining (I) and Western blotting (II). L7ΔN54 is a mutant of L7 that lacks the first 54 amino acid residues at the NH_2_-region of L7; N54 is a peptide that consists of the first 54 amino acid residues of L7. (B) Dot blotting assay using anti-L7n antibody. Each dot contains one OD_254_ unit of HeLa ribosome particles (80 S) or 50 µg of S100 fraction (**C**) Immuno-reactivity of anti-L7n antibody against the ribosome (TP80) and the cytosolic S100 fraction analyzed by SDS-PAGE with Coomassie blue staining (I) and Western blotting (II). Each lane was loaded with 50 µg each of TP80 or cytosolic S100. (D) A sucrose density gradient analysis of the 60 S dimers (marked by arrow) from the reaction mixture after incubation of the 60 S subunit with anti-L7n. (E) Electron microscopy images of the 60 S immune-complexes. Bar = 20 nm.

In this study, an anti-L7n antibody against an ES peptide that consists of the NH_2_-terminal 54 amino acid residues has been successfully generated (Fig. 1A). The antibody was first characterized as surface-specific against ribosomes and this was evident from the positive result of dot blotting assay (Fig. 1B). In parallel, Western blotting indicated that the antibody specifically reacted with L7 in the ribosome fraction prepared from HeLa cells, but not with the cytosolic S100 fraction (Fig. 1C), even when the S100 fraction was heavily loaded. The lacking of L7 protein in the cytosolic S100 fraction made it possible to use the anti-L7n antibody as a probe to locate cellular ribosome particles inside the cell *per se*.

In addition to the above, the ribosome-surface specificity of the anti-L7n antibody was further ratified by an immuno-electron microscopy (IEM) [16] where the formation of 60 S immune-complexes (60 S dimers) (Figs. 1D and 1E) was confirmed after incubation of the 60 S subunit with anti-L7n antibody.

### The cellular distribution of ribosome in cells at different phases of the cell cycle

**Figure 2:**
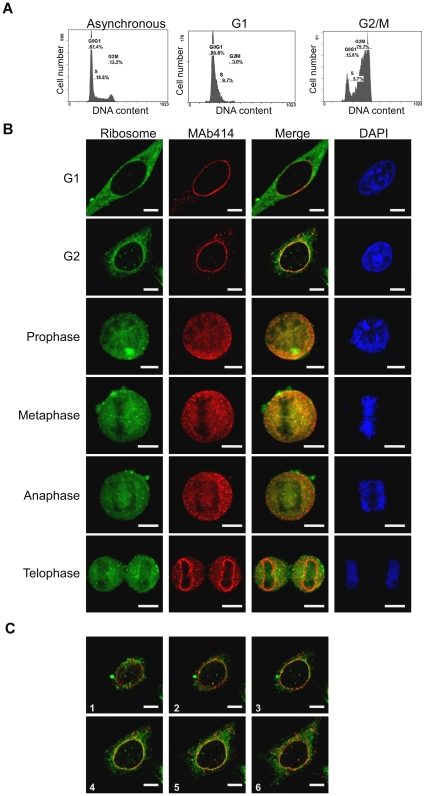
The ribosome distribution of synchronized HeLa cells during the cell cycle. (A) Cytometry measurement of the synchronized HeLa cells. (B) The cellular distribution of the ribosomes within HeLa cells during cell division was detected using anti-L7n, and co-stained with MAb414 antibody, which is specific to the nuclear pore complex. (C) A serial confocal microscopic section to show the ribosome gathering toward to the nucleus in the G2 cell. The spacing between sections was 1 µm. The representing picture is a merged image of ribosome and MAb414 staining. Bar = 10 µm.

After confirming the surface-specificity of the anti-L7n antibody as well as the non-detection of free L7 protein in the cytoplasm, the anti-L7n antibody was next used to probe the distribution of ribosomes in the synchronized cells using immunofluorescent staining. Accordingly, we first prepared synchronized HeLa cells by using the double thymidine block method. An 80–87 synchronize percentage was generally achieved (Fig. 2A), which helped the correct identification of the phase of the synchronized cells. Consequently, we carried out double immunostaining using anti-L7n antibody and MAb414 antibody, which immune-interacted with the nuclear pore complex. This revealed the relationship between ribosome distribution and the nuclear envelope during each phase of the cell cycle. In G1 cells, there was an even distribution of fluorescence due to ribosome staining throughout the cytoplasm (Fig. 2B). During G2, the cells displayed increased fluorescent staining around the edge of the nucleus (the nuclear envelope) (Figs. 2B and 2C). At the M phase, including prophase through metaphase and late anaphase, a similar cytoplasmic dispersion pattern to G1 (Fig. 2B) was found. Moreover, when the fluorescent intensity of each daughter cell during late telophase (Fig. 2B) was measured, the ratio was close to one (0.99). This suggested that each daughter cell appears to show similar staining of ribosome particles.

### Electron microscopic examination of the cellular distribution of ribosome in G1 and G2 cells

**Figure 3:**
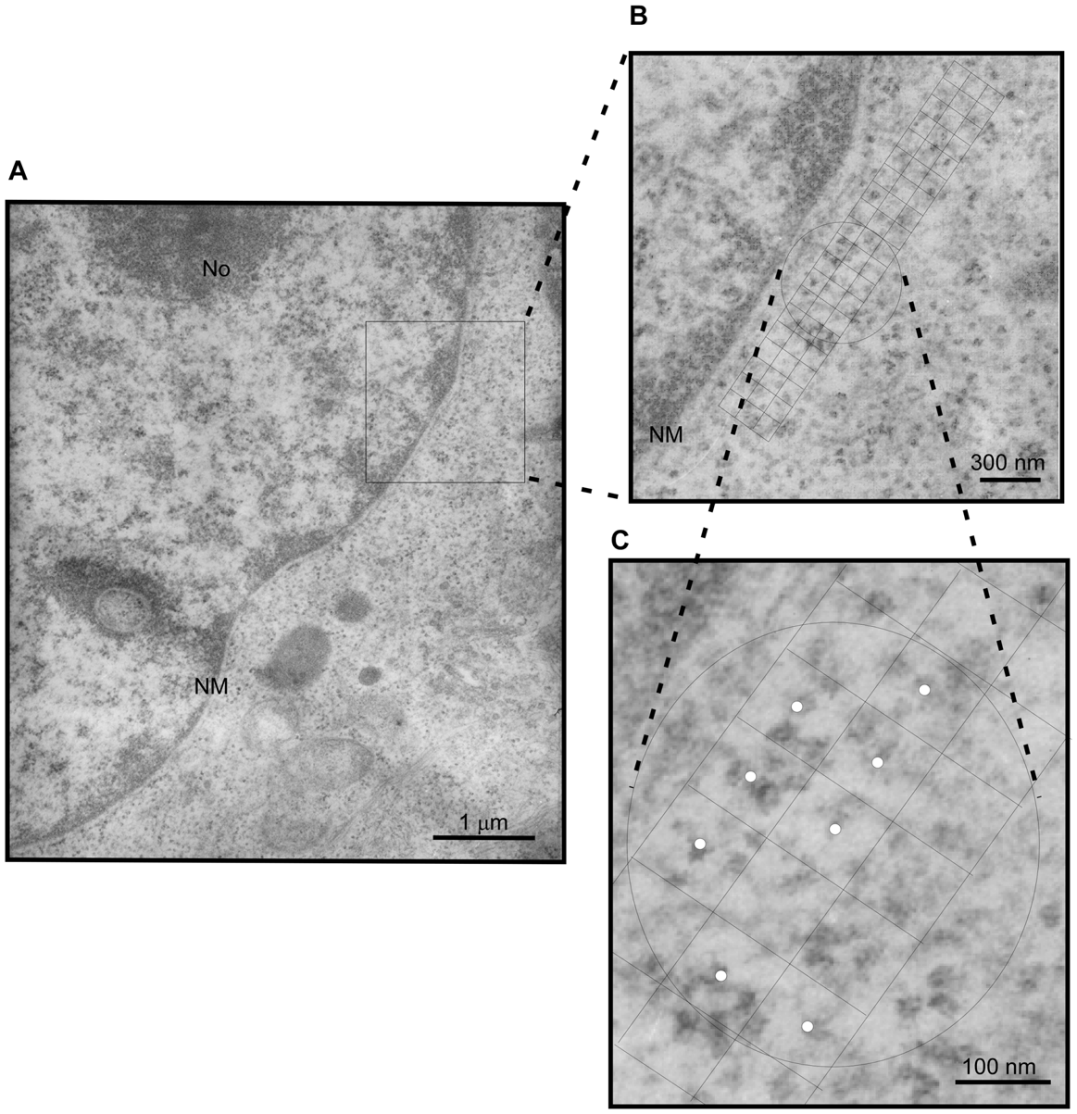
Morphometric analysis of the ribosome population in the G1 and G2 phases. (A) Example of a higher magnification view of a G1 cell with a mounted triple-lattice grid for counting the density of ribosomes. (B) An expanded view shows how the grid was mounted on a region proximal to the nuclear envelope. (C) An enlarged view shows the exact co-localization of the ribosome particle on the center white dot of the grid that represents one positive score. NM, nuclear membrane; No, nucleolus.

The observation that ribosomes gather toward the nuclear envelope in G2 cells is rather peculiar, and required further investigation. Thus, an examination at electron microscope level was carried out. Accordingly, electron micrographs of G2 and G1 cells were prepared (Fig. S1), and ribosome particles were ready identified at high magnification (72,000 from ×24,000 negatives). Consequently, the distribution of ribosome particles was analyzed using a morphometric assay [17]. The assay was executed in two assigned regions. One was selected as close to the nucleus (a 500 nm wide zone around the nuclear membrane) and the other was well away from the nucleus (approximately 1000 nm or further away from the nuclear membrane). A good example of the execution of the assay is given in Figure 3. In this study, micrographs of two hundred G1 and two hundred G2 cells were analyzed and the results (Table 1) were subjected to a paired t test. The results of the t test for G1 phase cells showed that t<t_0_, indicating that there is no significant difference with respect to the ribosome population *vs* their location. However, for the G2 phase cells test, t>t_0_ value, which confirms that the ribosome population is indeed denser in the area proximal to the nuclear envelope compared to an area distal to the nucleus.

**Table 1:** Paired t test for the distribution of ribosomes in G1 and G2 phase of cells.

Phase of cell cycle	Ribosomes		Di	Di^2^	Σ (NM+CM)
	Near NM	Near CM			
G1	17.96875	18.9375	0.96875	28.90625	32.90625
G2	23.04167	12.6225	10.47917	13.74375	35.60417

NM represents nuclear membrane; CM represents cytoplasm membrane.

SSD (sample standard deviation) = √[Σ(Xi−X)2/n−1] = Σ Di^2^−(Σ Di)2/n.

t = D/√[SSD/n(n−1)].

### Co-localization of ribosome with different nuclear membrane marker proteins during M phase

**Figure 4:**
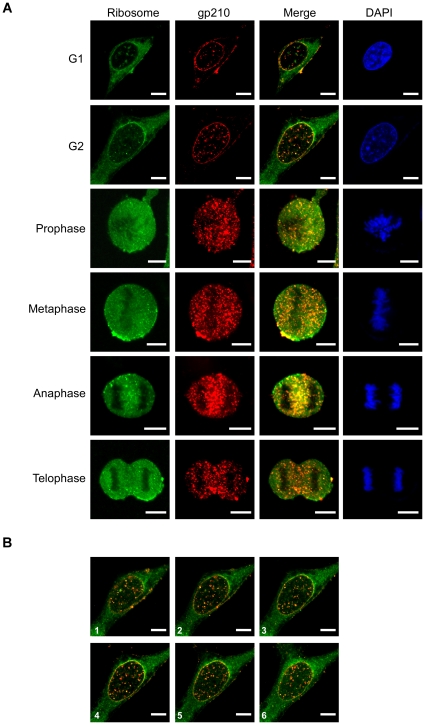
Co-localization of ribosome particles with the nuclear regulator protein gp210. (A) The cellular distribution of the ribosomes within the HeLa cells during cell division was detected using anti-L7n, and co-stained with anti-gp210 antibody, which is specific to the nuclear membrane. (B) A serial confocal microscopic section to show the ribosome gathering toward to the nucleus in the G2 cell. The spacing between sections was 1 µm. The representing picture is a merged image of ribosome and gp210 staining. Bar = 10 µm.

**Figure 5:**
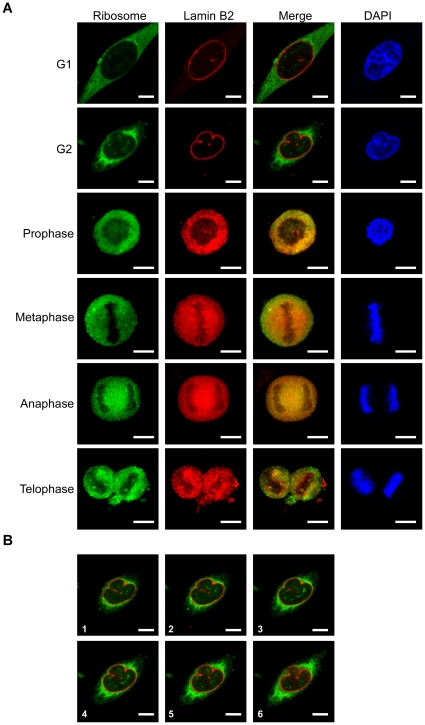
Co-localization of ribosome particles with the inner nuclear lamin B2. (A) The cellular distribution of the ribosomes within the HeLa cells during cell division was detected using anti-L7n, and co-stained with anti-lamin B2 antibody, which is specific to the nuclear inner membrane. (B) A serial confocal microscopic section to show the ribosome gathering toward to the nucleus in the G2 cell. The spacing between sections was 1 µm. The representing picture is a merged image of ribosome and lamin B2 staining. Bar = 10 µm.

The ribosome gathering toward the nuclear envelope at G2 phase suggests a possible relationship might exist between the ribosome and the membrane of nuclear envelope during the cell cycle, since the membrane of the nuclear envelope is disassembled, and they reassemble as the cells proceed from the G2 to the M phase. To clarify such a possibility, we further carried out the co-localization examination of ribosomes with additional nuclear membrane protein markers during M phase. The markers used included a nuclear integral membrane protein gp210 [18], and an inner membrane protein lamin B2 [19]. The results were rather disappointing, since the distribution pattern was identical to that of the nuclear pore complex [20] as shown above. None of these nuclear envelope markers were found to co-distribute with the ribosomes during the disassembly and re-assembly of the membrane of the nuclear envelope (Figs. 4 and 5). This implies that the mechanism of ribosome segregation, if there is one, would seem to be independent of the processing of disassembly and reassembly of the nuclear envelope.

### Effect of ER transformation on ribosome distribution during the cell cycle

**Figure 6:**
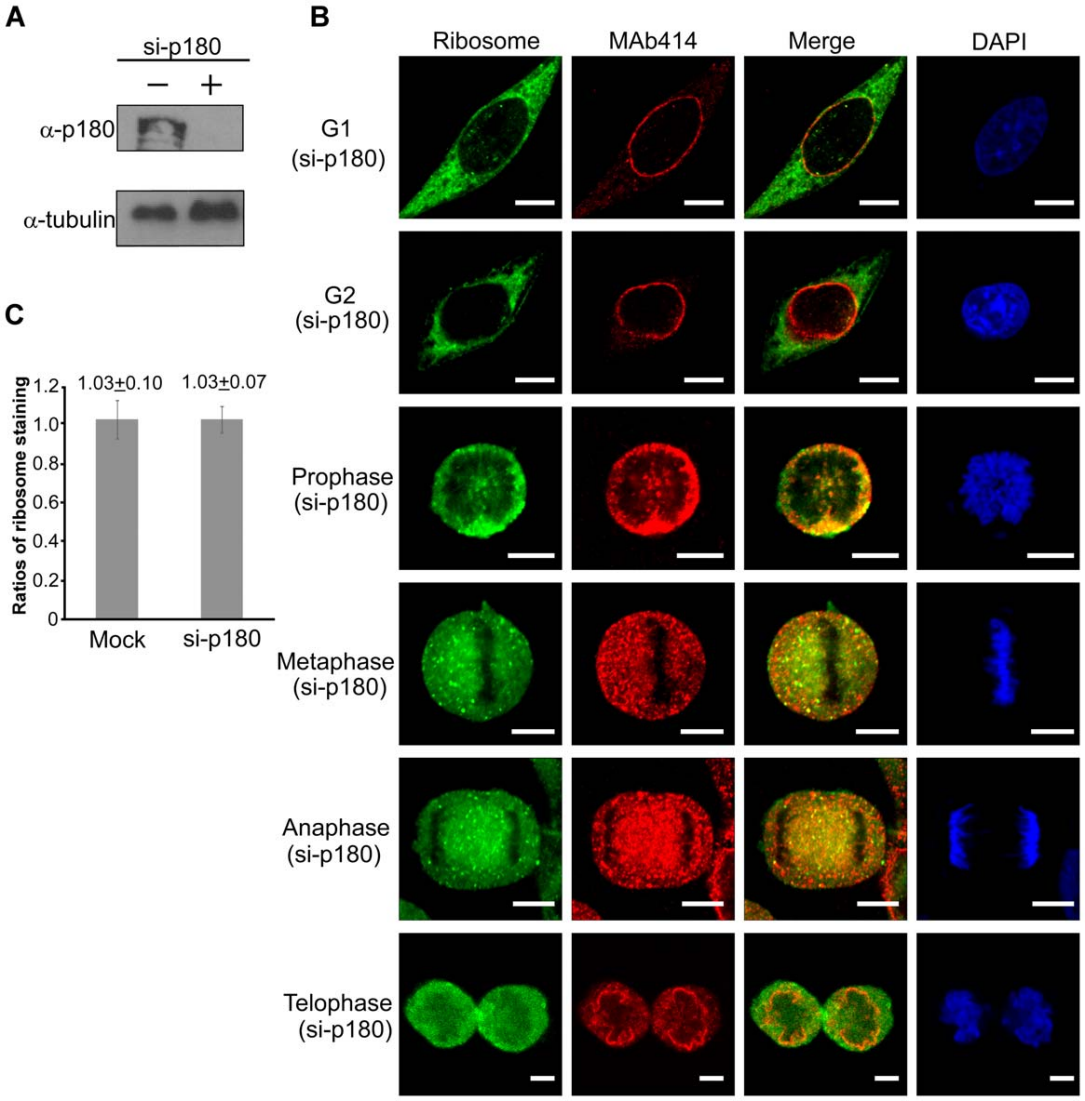
The ribosome distribution in HeLa cells under the p180 knock-down. (A) siRNA knock-down of the expression of p180. (B) The cellular distribution of the ribosomes within HeLa cells that had been down-regulated with respect to expression of p180 during cell division as detected by anti-L7n and MAb414 antibodies. Bar = 10 µm. (C) The ratio determined from the fluorescent intensity in each paired daughter cell during late telophase, stained by anti-L7n antibody. Ratio of mock was obtained from the non-siRNA knock-down cells.

The ER membrane is known to mediate ribosome binding [15], [21], and as an extension part of the nuclear membrane, it undergoes an ultra-structural transformation during the disassembly and reassembly of the nuclear envelope [21]. Therefore, we investigated the effect of changes in the ER on ribosome distribution. Accordingly, we attempted to down-regulate one of the major proteins in the ER membrane, a membrane integral protein p180, which is known to mediate ribosome binding [22], [23], and then determined whether or not this disturbance to the ER structure would or would not affect the distribution of ribosome during the cell cycle. Using a siRNA approach, we down-regulated the expression of p180 protein (Fig. 6A), and examined the cellular distribution of ribosomes in treated cells. The result of observation (Fig. 6B), using anti-L7n, and MAb414 antibodies, showed that there was little change in the ribosome distribution pattern (Fig. 6B), except that the reassembled nuclear envelope appeared to have a distorted ruffled shape as the cells reached late telophase (Fig. 6B). However, when the fluorescent intensity of each daughter cell during the late telophase was examined, there seemed to be no effect, because the ratio close to one (1.03) (Fig. 6C) was again found for the two daughter cells.

## Discussion

How ribosomes become evenly distributed into the two daughter cells at the end of cell division has never been characterized. The question is biologically important because ribosomes are the power houses of protein synthesis and have a major physiological impact on the future daughter cells, which heavily depend upon the pre-existing ribosomes to maintain early homeostasis [3], [8]. This is simply because it takes time to synthesize a new ribosome. Moreover, it has recently been reported that the ribosome biogenesis is sensed at the start cell cycle checkpoint [1], and this suggests that dividing cells have a mechanism that handles intracellular ribosome distribution to daughter cells. This idea was put forward many years ago [9]. In these circumstances, determining the ribosome distribution patterns in cells at different phase of the cell cycle may provide clues about how ribosome segregation is controlled. For this reason, we have generated a surface-specific antibody against ribosome particles by raising an antibody against a eukaryotic ES peptide derived from the first 54 amino acid residues at the NH_2_-region of L7. This area was chosen because the ES has been proposed to be exposed on the ribosome surface [12], [13]. Consequently, the surface specificity of the antibody was confirmed by antibody's immuno-reactivity to the ribosome particles, and more perusable by showing the formation of 60 S immune-complexes (60 S dimers) using an immuno-electron microscopic technique. Such a demonstration of using an ES peptide to create a ribosome surface antibody provides a good way of characterizing the ES study involving the structure and function of eukaryotic ribosomes.

Using the surface properties of the anti-L7n antibody, we were able to gain new insights into how ribosomes are distributed in the cells at different phases of the cell cycle. We found that ribosomes were dispersed across the cytoplasm of the cell during all phases of the cell cycle except during G2 phase. In G2 phase, the ribosomes showed a tendency to gather near the nuclear envelope. These general findings on the broad cytoplasmic dispersion pattern were understandable; however, the uniqueness of the nuclear gathering pattern in G2 demonstrated by immunoflourescent staining, and confirmed by electron micrographs, was unexpected. Nevertheless, if this gathering event is considered, this leads to the question as to whether the phenomenon is important. Perhaps, the event has truly physiological meaning and might represent a mechanism that allows ribosome to be more evenly distributed between the daughter cells. One might speculate that the ribosomes might take a ride on the nuclear membrane during the disassembly/assembly process of the cell division. Exploring this possibility, we carried out several co-localization experiments to detect any possible relationship between the ribosomes and nuclear envelope markers. However, when the co-localization of the ribosomes with membrane markers that cover most parts of nuclear envelope, namely the nuclear pore complex [20], the nuclear membrane protein gp210 [18], and the inner nuclear membrane protein lamin B2 [19], were examined, we were unable to find any relationship during the process of cell division. This suggests that any mechanism involved in ribosome segregation is likely to be independent of the events of nuclear envelope disassembly and re-assembly. In addition, by knock-down of the expression of the integral membrane p180 protein, we found that disturbance of the ER did not affect the even segregation of ribosomes into daughter cells. Together the fact, we speculate that the mechanism of ribosome segregation is independent of intercellular exchange with various membranes despite the fact that these membranes show a high affinity for ribosomes [24]. However, we as yet are unable to put forward a mechanism that explains the even segregation of ribosomes into daughter cells, notwithstanding the fact that we know that the ribosome do move towards the nuclear membrane during G2 phase. Nevertheless, there have been recent studies using nanoparticles to investigate dynamic movement within the cytoplasm during cell division [25], [26]. These studies indicate that an over-dispersed Poison probability distribution occurs. Partitioning of internalized nanoparticles during cell division seems to be random and asymmetric, following a binomial distribution with a mean probability of 0.52–0.72 [27]. Our measurement of the ribosome particles received by the daughter cells falls within the range. We believe that the segregation of ribosome is an ordered stochastic process but far from random, in the manner of nanoparticles inside the cell, and the process involves a strong tendency toward equality as suggested earlier [9]. The significance of this study is the first ever to report on how ribosome, a non-membrane-bounded organelle, is distributed during cell cycle.

## Materials and Methods

### Generation of a surface-specific antibody against L7 and the ribosome particle

The ES peptide that covers the first 54 amino acid residues of the NH_2_-terminal region of human ribosomal protein L7 has been determined previously [13], [15] and this was used as the antigen to prepare a polyclonal antibody, designated as anti-L7n, and it was produced by a commercial company (Abcam, Taiwan). Antibodies against gp210 (sc-79500, Santa Cruz), lamin B2 (ab8983, Abcam), and the nuclear pore complex MAb414 (MMS120P, Covance), which targets the nuclear membrane, were purchased commercially.

### Characterization of the ribosome surface specificity of the anti-L7n antibody

The surface-specific nature of the anti-L7n antibody against ribosome was first examined using a dot blotting assay as previously described [13]. Briefly, one OD_254_ unit of ribosomes and 50 µg of S100 fraction proteins, which had been prepared from HeLa cells (American Type Culture Collection, ATCC CCL2) were dissolved in a TKM buffer containing 20 mM Tris-HCl pH 7.6, 50 mM KCl, and 3 mM MgCl_2_, and spotted with a diameter of 2 mm onto a nitrocellulose (NC) membrane. The membrane was blocked with TKM buffer containing 5% BSA before being subjected to detection by anti-L7n. Positive immuno-reactivity was found by phosphoimaging. In parallel, the ribosome and S100 fractions were further analyzed by SDS-PAGE, followed by Western blot detection using the anti-L7n antibody.

### Immuno-electron microscopy (IEM)

To further confirm the surface specificity of the anit-L7n antibody, we carried out IEM. This was done by examining the formation of the 60 S immune-complex, which is a 60 S dimer linked by an anti-L7n antibody. In brief, 60 S subunits isolated from HeLa cells were incubated with anti-L7n antibody under the conditions previously described in earlier IEM studies of the *E. coli* ribosome [16]. The reaction mixture was then analyzed by ultracentrifugation using a 10–30% sucrose density gradient in TKM buffer, and the peak corresponding to the 60 S immune-complex (60 S dimer; approximately 110 S) was collected and directly loaded onto an electron microscopic grid. After 2% uranium acetate staining, the grid was observed by a standard EM protocol on a JEM-1230 electron microscopy at an accelerating voltage of 80 kV.

### Synchronization of HeLa cells and preparation of mitotic cells

The preparation of the synchronized cells was carried out using the established double thymidine block protocol. In brief, HeLa cells were maintained in medium A containing DMEM (Dulbecco's modified Eagle's medium, GIBCO™) supplemented with 3.7 g/l NaHCO_3_, 10% fetal bovine serum, 1% glutamate, and 1% streptomycin under air/CO_2_ (19∶1) at 37°C in a humidified incubator. When the cells reached the exponential phase, 2 mM thymidine was added to the culture to arrest the growth. After 24 h of treatment, the cells were washed with DMEM without fetal bovine serum to remove excess thymidine, and allowed to resume its growth in medium A for another 12 h. At this point the thymidine treatment, washing, and returned to medium A were repeated once again in order to achieve synchronization. Synchronization of the cells was examined at 0 h, 8 h, 12 h, and 16 h using a Beckman Coulter FC500 (Beckman Coulter, Taiwan).

### Immuno-fluorescence observation of the cellular distribution of ribosome

Indirect fluorescent detection was carried out using the anti-L7n antibody as the primary antibody. Synchronized HeLa cells that has been grown on a coverslip were washed with PBS three times and fixed in acetone at room temperature for 15 min. Antibody was added and incubated in a moisture chamber for 15 min. After extensive washes, a second antibody, anti-rabbit IgG conjugated with FITC (sc-2012, Santa Cruz), was incubated with the cells for another 30 min. This was followed with washes in PBS and buffered glycerol (glycerol∶PBS = 9∶1) washes. Fluorescence was excited using a laser beam (568 nm wavelengths) and observed by laser confocal microscope (FV1000, Olympus).

### Preparation of electron micrographs of the synchronized cells using the morphometric assay

HeLa cells at different stages of the cell cycle were collected and fixed in 0.1 M phosphate buffer containing 3% glutaraldehyde for 30 min. The cell pellet was washed with 0.1 M phosphate buffer and post-fixed with 0.1 M osmium tetrooxide in PB for 1 h, dehydrated with graded ethanol, and then embedded in Epon 812. A 60–90 nm thin sections were prepared, and were stained with 2% aqueous uranyl acetate for 10–20 min. All micrographs were taken on a JEOL JEM 2000EXII electron microscope. Magnifications were determined by means of a carbon-grating replica.

Two magnifications, ×10,800 and ×72,000, were prepared. The lower magnification was used to define the morphology of cells that had been arrested at different phases; while the higher magnification was used for localizing the ribosome population density. The morphometric assay was carried out on electron micrographs of HeLa cells at the higher magnification of ×72,000 (from ×24,000 negative). Examined micrographs are shown from 5 different cell blocks.

A modification of the morphometric analysis method designed by Mori and Christensen [17] was adapted to quantitatively measure the distribution of ribosomes on an electron micrograph. One hundred micrographs at high magnification were analyzed. The analysis was carried out by placing a transparent overlay bearing a triple-lattice grid (3×20 grids) that had been added to the electron micrograph. Each grid covered an area of 100×100 nm. A total of 3×20 grids with an area of 6×10^5^ nm^2^ were used. Each grid has a mark at the center. For a positive score, the presence of a ribosome particle on the grid must coincide with the mark. Only one score was counted for each grid regardless of how many ribosomes might be presented on the grid. Under these guidelines, two regions of the density of ribosomes were taken to measure: one was a 500 nm wide zone around nuclear membrane, the other was distal to the nucleus (approximately 1000 nm or further away from the nuclear membrane). The results for each cell phase were analyzed using a paired t test.

### siRNA knock-down of an integrated membrane protein p180 in mitotic cells

Based on a recent study of p180 [23], siRNA duplexes (1299003, Invitrogen) were used to down regulate the expression of p180 gene. In brief, lipofectamine™ 2000 (11668, Invitrogen) was used to transfect siRNA duplexes (100 nM) into HeLa cells in serum and antibiotic-free DMEM according to the manufacturer's protocol. After the knock-down treatment, cells were subjected to arrest at various mitotic stages as described above. The knock-down efficiency was checked at 48 h post-transfection by Western blotting using anti-p180 antibody (ab95983, Abcam).

## Supporting Information

Figure S1 **Electron micrographs of HeLa cells at different phase of cell cycle.** The representative electron micrographs are (A) a cell at the G1 phase; (B) a cell at the G2 phase; (C) at the beginning of metaphase (prepared from mitotic cells). Magnifications are ×10,800 (from ×3,600 negative). NM, nuclear membrane; No, nucleolus; M, mitochondria. Bar = 2 µm. The G1 cell shows a characteristically enlarged rough-shaped nucleus with a centered nucleolus (A); the G2 cell has a smaller elongated form of the nucleus with its nucleolus slanted toward the nuclear envelope (B); the cell at the beginning of M phase shows a dissolved nucleus and the heavy electron dense materials has become less stained (C) which is characteristic of this phase.
(TIF)
