# Supplementary figures and images for: Ribosome Distribution in HeLa Cells during the Cell Cycle
Source: PLoS One. 2012 Mar 5;7(3):e32820. doi: 10.1371/journal.pone.0032820 (PMC3293906; doi:10.1371/journal.pone.0032820)

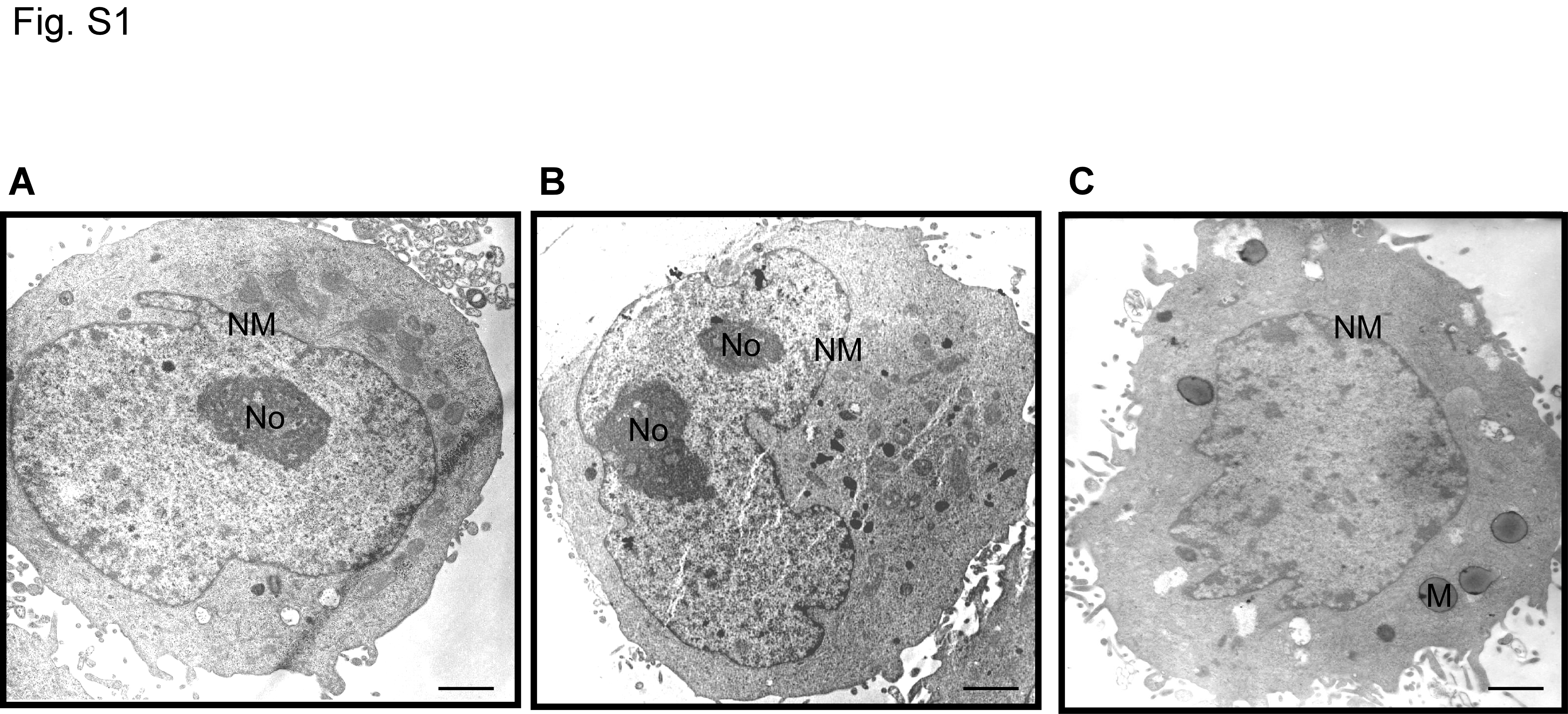

Supplement: Figure S1 — Electron micrographs of HeLa cells at different phase of cell cycle. The representative electron micrographs are (A) a cell at the G1 phase; (B) a cell at the G2 phase; (C) at the beginning of metaphase (prepared from mitotic cells). Magnifications are ×10,800 (from ×3,600 negative). NM, nuclear membrane; No, nucleolus; M, mitochondria. Bar = 2 µm. The G1 cell shows a characteristically enlarged rough-shaped nucleus with a centered nucleolus (A); the G2 cell has a smaller elongated form of the nucleus with its nucleolus slanted toward the nuclear envelope (B); the cell at the beginning of M phase shows a dissolved nucleus and the heavy electron dense materials has become less stained (C) which is characteristic of this phase. (TIF) [file pone.0032820.s001.tif]
